# Eight Years of the Great Influenza Survey to Monitor Influenza-Like Illness in Flanders

**DOI:** 10.1371/journal.pone.0064156

**Published:** 2013-05-17

**Authors:** Yannick Vandendijck, Christel Faes, Niel Hens

**Affiliations:** 1 Interuniversity Institute for Biostatistics and statistical Bioinformatics, Hasselt University, Diepenbeek, Belgium; 2 Centre for Health Economic Research and Modeling Infectious Diseases, Vaccine and Infectious Disease Institute, University of Antwerp, Wilrijk, Belgium; Melbourne School of Population Health, Australia

## Abstract

In 2003, an internet-based monitoring system of influenza-like illness (ILI), the Great Influenza Survey (GIS), was initiated in Belgium. For the Flemish part of Belgium, we investigate the representativeness of the GIS population and assess the validity of the survey in terms of ILI incidence during eight influenza seasons (from 2003 through 2011). The validity is investigated by comparing estimated ILI incidences from the GIS with recorded incidences from two other monitoring systems, (*i*) the Belgian Sentinel Network and (*ii*) the Google Flu Trends, and by performing a risk factor analysis to investigate whether the risks on acquiring ILI in the GIS population are comparable with results in the literature. A random walk model of first order is used to estimate ILI incidence trends based on the GIS. Good to excellent correspondence is observed between the estimated ILI trends in the GIS and the recorded trends in the Sentinel Network and the Google Flu Trends. The results of the risk factor analysis are in line with the literature. In conclusion, the GIS is a useful additional surveillance network for ILI monitoring in Flanders. The advantages are the speed at which information is available and the fact that data is gathered directly in the community at an individual level.

## Introduction

For healthcare workers, a timely and accurate system to monitor the spread of seasonal and pandemic influenza in the general population is important. Almost every influenza epidemic is associated with an increase in hospitalizations and excess deaths [1], [2]. Because infection with the influenza virus is difficult to diagnose without virological confirmation, the best surveillance indicator of influenza in the community is the incidence of influenza-like illness (ILI). ILI is defined as an illness with symptoms similar to an influenza infection.

Traditional surveillance systems rely on clinical and virological information from ILI patients that visit their physician. In Belgium, this system is organized by the Scientific Institute of Public Health and is based upon ILI consultations at general practitioners (GPs) that participate in the Belgian Sentinel Network [3]. During an influenza season, these data are published online on a weekly basis by the European Influenza Surveillance Network (EISN). Due to the ever increasing usage of the internet, internet-based monitoring systems have been set up as well, such as: (*i*) surveillance based on voluntary participation in an online survey [4]–[7], and (*ii*) surveillance based on ILI-related queries entered at online search engines [8]–[12].

In the Netherlands and Belgium, internet-based monitoring of ILI via an online survey was implemented during the 2003–2004 influenza season and is known as *De Grote Griepmeting* or the *Great Influenza Survey* (GIS). The main objective of the GIS is to rapidly asses the ILI incidence level in the community. The penetration of the GIS in Belgium is, in practice, limited to the Flemish part of Belgium because of its implementation in Dutch. Several years later, similar surveys were initiated in Portugal, Italy and the United Kingdom, while it was additionally implemented in five other European countries in 2011. This project of internet-based monitoring of ILI across different European countries is known as *Influenzanet* and creates a uniform system that allows for the direct comparison of ILI rates between these countries [13]. Studies of the Dutch GIS showed that the young and the elderly are underrepresented in their survey. Nevertheless, excellent correlations between the estimated incidences from the GIS and those obtained from the sentinel network were observed [4], [5]. Similar correlations were found for the 2006–2007 Belgian influenza season [6]. High correlations were also observed between the ILI trend based on the UK flu survey monitoring system and the trend as reported by GPs during the pandemic influenza season in 2009–2010 [7].

Google Flu Trends is another recently developed surveillance system and is based on internet search queries related to ILI. For example, some search query topics are influenza complications, cold/flu remedy and antibiotic medication. The estimates of ILI activity are provided online in near real time [11]. Trends observed in Google Flu Trends correlate well with trends reported by traditional surveillance systems [12].

This study aims to assess the validity of the GIS in Flanders with respect to the trend estimation of ILI incidence and the representativeness of the survey population. The study covers surveys from the 2003–2004 to 2010–2011 seasons. The validity of the GIS is studied by comparing estimated ILI trends with the recorded ILI incidence of (*i*) the Belgian Sentinel Network and (*ii*) the Google Flu Trends. To further validate the usability of the survey, a risk factor analysis is conducted to investigate whether risk factors in the survey population are in line with those reported by the ILI literature.

## Materials and Methods

### Design of the GIS

The GIS is based on the voluntary participation of individuals in an internet survey. An individual can join the survey at any time. At registration, an intake questionnaire must be completed, containing demographical, medical and lifestyle questions. Participants are asked weekly by email to complete a symptom questionnaire documenting any symptoms experienced since their last visit. Participants also record their highest body temperature (if measured) and whether a fever was observed with or without sudden onset. If symptoms and/or fever are reported, the onset date is asked. Participants can indicate whether they consulted a GP. Finally, it is asked whether the symptoms experienced led to a change in the participant’s daily behavior. Further details can be found in Marquet *et al.* [4].

To define ILI, the following ILI case definition is used, which closely resembles the WHO guidelines [14]: *A sudden onset of fever, namely, a measured body temperature of* 38°C *or more, accompanied with headache or muscle pain and accompanied with cough or a sore throat*. The date of fever onset is used as the date of ILI onset.

The data of the Flemish GIS can be obtained upon request via the *Influenzanet* website [13].

### Sample Used in the Analysis

To reduce the effect of volunteers that only participated rarely and those who took part as a one-off response to their current symptoms, data from the first symptom questionnaire are excluded and only data of participants that completed at least three symptom questionnaires are used [13]. We will refer to this sample as the *restricted sample*. A comparison between the *complete* (no data excluded) and the *restricted sample* is made. Other restriction have been proposed in the literature, albeit with few additional insights [4], [5], [7].

When a participant experienced ILI in two or more symptom questionnaires that are not separated by more than a fortnight, these symptoms are considered to belong to the same ILI episode.

### Representativeness of the GIS Population

To investigate the representativeness of the GIS population, demographic and medical statistics from the Flemish population are compared to the corresponding numbers obtained from the GIS using the *complete sample*. Similar results are obtained if the *restricted sample* is used.

Age, gender and spatial distributions are compared between the two populations. The spatial distribution is calculated by the population proportion per province. The Flemish population statistics are obtained from Statistics Belgium [15]. The prevalence of asthma and the prevalence of diabetes in the Flemish population are compared with the self-reported conditions in the GIS population. Influenza vaccine coverage for the total population and persons older than 65 years are also compared between the two populations. The prevalence statistics and vaccination coverage in the Flemish population are obtained from the Health Interview Survey (HIS) of the years 2001, 2004 and 2008 [16]–[18]. The HIS is a large-scale health survey in Belgium that is held every three to four years.

### Validity of the GIS

ILI incidence per week is estimated by the number of GIS participants with ILI onset in a certain week divided by the number of active participants in that week [6] (referred to as the *model-free* estimation approach). Note that the numerator is constructed based on the onset of ILI, and not on the week that the participant filled in the symptom questionnaire. A participant is considered to be active between the day of the completion of their first symptom questionnaire and the day of the last completed symptom questionnaire. A participant who is active for a whole week is counted as one person-week, while, for example, a participant who completed their last symptom questionnaire on Wednesday, is counted as 3/7 person-weeks for that particular week. Several issues arise when using the *model-free* estimation approach to obtain ILI incidence trends: (*i*) rough trends are obtained; (*ii*) not all data are used at once, since the incidence estimate of a certain week does not take into account the incidence estimates of the other weeks; and (*iii*) no estimates of variability around the estimated trends are obtained. To overcome these issues, a random walk model of first order (referred to as the *RW1-model*) is used to estimate ILI incidence trends [19], [20] (see Text S1). This model allows one to estimate a smoother trend because it is able to separate a trend from the noise. Associated variability bands are also obtained. In contrast, the *model-free* method implicitly assumes that there is no variability in the data.

The estimated ILI incidence trends are compared with the trends of (*i*) the Belgian Sentinel Network and (*ii*) the Google Flu Trends. These data are publicly available [21], [22]. The Sentinel data are not available on the Flemish level, and therefore, the Belgian data are used as a proxy. To determine the Google Flu Trends incidence in Flanders, we consider a weighted sum of the incidences of the five Flemish provinces, in which the weights are chosen according to the population proportions of these provinces. For the GIS to be a valid tool for ILI surveillance, estimated trends from the GIS should coincide with the trends of the other two networks [23]. Pearson correlation coefficients are used to measure this coincidence. Additionally, we examine whether there is a better association between the two networks when a time lag is taken into account.

To further validate the GIS, risk factors for ILI are estimated. The relative risks (RR) for several covariates are estimated based on a multivariate regression model. The probability that an individual has influenza during a particular season is modeled as a function of several covariates of interest. The covariates considered are age, gender, living with children, means of transportation, chronic diseases (asthma and diabetes), allergies, smoking status, physical activity and having pets. We control for the influenza vaccination status in the model. Participants are considered to be vaccinated in a particular season if they reported having received an influenza vaccine during any stage of the season. The risk factors are estimated simultaneously for the influenza seasons from 2003 to 2011, without the pandemic H1N1 2009–2010 season which is analyzed separately. Season is introduced as a covariate in the model to capture a season-specific difference in the risk of acquiring ILI. An interaction between influenza vaccination status and season is considered, as the effectiveness of an influenza vaccine changes from season to season. Since ILI is a relatively common disease, logistic regression models are not appropriate to estimate RRs, as the RR in such a case is not well approximated by the odds ratio (OR) [24]. Poisson regression with robust standard errors is used to obtain directly valid estimates of the RRs [25].

### Ethics Statement

The data were de-identified and analyzed anonymously. The study was carried out according to the Belgian legislation on privacy. Participation was carried out via registration on the website www.degrotegriepmeting.nl. People who registered were invited by weekly emails to participate in an online questionnaire. Participants are able to refuse further participation at any time after registration by not completing the online questionnaire. Note that there was no physical or psychological intervention to which participants were exposed in this study; therefore, it was an observational study for which no informed consent is necessary according to the Belgian legislation of May 7, 2004: “Wet inzake experimenten op de menselijke persoon” (“Law on experiments involving the human subject”; Article 8, 2°; Article 3, 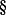
1).

## Results

During the eight influenza seasons under study, 19263 individuals participated in the GIS during one or more season. Almost half of them (46.75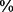) participated only in one season, 24.54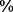 participated in four or more seasons, and 1.87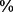 participated in all eight seasons. Some results on the GIS are presented in Table 1. The highest number of participants is observed in 2005–2006. In the first two seasons, the number of participants and the mean number of reports per week are the smallest. In the last three seasons, the smallest percentages of volunteers that participated only once during a season and the largest percentages of individuals that participated at least three times are observed. About 80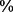 (77.98–88.15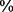) of the participants stayed home, and about 60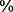 (53.16–70.76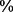) went to their GP when they reported an ILI episode. Of those that participated no more than two times in a season, the proportion of participants that experienced ILI is, in most seasons, lower than the overall proportion with ILI. However, the proportion of ILI episodes observed in the first symptoms questionnaire of a participant is higher than the overall proportion (except in 2008–2009). This result indicates that some volunteers start participating in the GIS as a response to their symptoms, but non-regular participation does not lead to higher ILI rates.

**Table 1:** Results on the Great Influenza Survey (GIS) from 2003–2004 to 2010–2011.

Characteristic	2003–04	2004–05	2005–06	2006–07	2007–08	2008–09	2009–10	2010–11
week numbers included	45–14	47–18	45–17	48–18	44–18	44–27	28–21	44–18
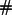 participants	3135	2084	11579	6862	8678	4977	5682	4551
# weekly reports	24314	30119	148246	100446	139924	134503	159251	83525
mean number of reports/week	1105	1255	6177	4566	5382	3843	3462	3213
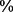 with ILI	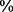 12.31	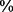 8.54	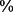 5.44	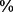 7.02	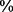 6.19	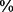 6.93	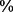 6.37	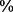 5.12
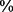 participated once	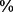 24.82	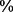 11.28	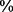 17.27	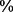 9.24	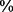 9.45	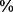 2.60	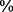 5.10	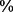 6.77
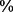 participated 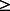 3 times	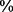 67.81	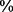 85.08	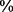 75.56	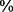 86.36	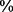 85.48	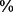 95.95	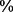 92.05	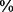 89.65
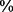 of ILI staying home	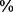 82.67	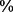 88.15	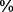 79.97	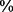 85.39	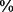 84.25	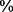 86.18	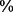 78.87	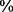 77.98
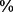 of ILI visiting GP	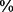 65.57	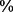 67.77	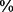 62.37	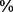 67.48	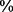 63.91	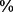 70.96	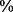 53.16	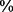 61.73
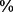 ILI, participated 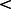 3 times	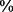 14.27	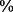 4.84	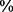 3.39	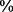 6.09	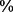 3.97	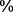 3.00	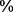 3.54	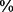 3.82
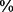 ILI in first symptom ques.	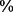 67.21	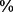 19.90	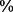 25.81	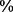 15.65	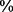 16.06	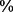 6.32	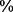 11.33	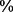 12.27

### Representativeness of the GIS Population

**Figure 1:**
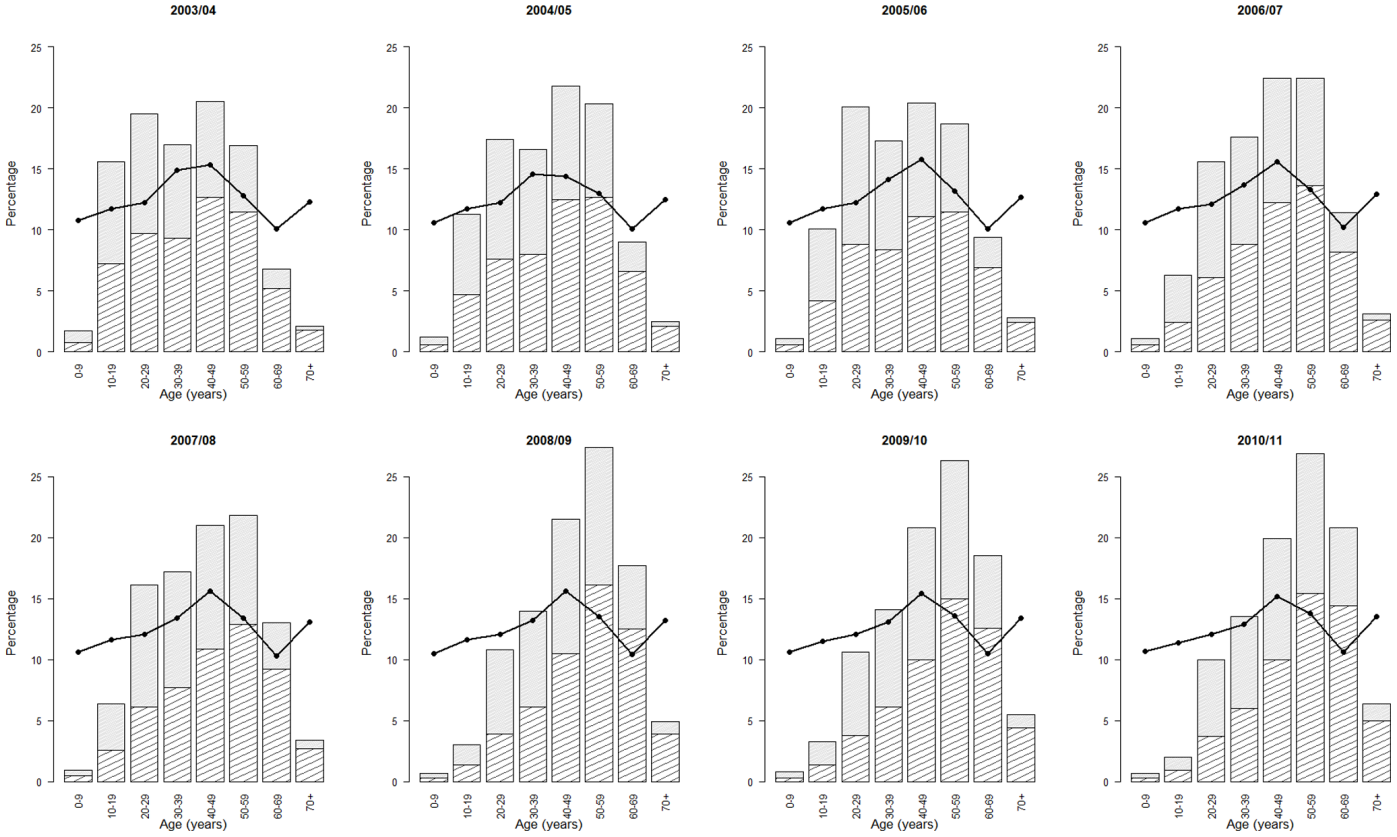
Age distribution of the GIS and Flemish population. For all eight seasons, the age distribution of the GIS population is presented in the histogram, in which the shaded area represents males and the full area represents females. The full line represents the age distribution in the Flemish population.

From Figure 1, it is clear that the GIS population is not representative in terms of age. The age group 0–9 years is underrepresented in all seasons (e.g., 53 participants of 0–9 years in 2003–2004 and 32 in 2010–2011). The 70+ age group is also underrepresented. However, an increase in participation, especially in males, is observed over the years (e.g., 66 participants aged 70+ in 2003–2004 and 291 in 2010–2011). A decline in participation is observed for the 10- to 19-year age group. Mainly, individuals between 30 and 69 years of age participate in the GIS.

There is an overrepresentation of males in the GIS population (Table 2), since the male-female ratio in the Flemish population is 49/51. Good similarities are observed between the prevalence of asthma and diabetes, in which the prevalence is sometimes slightly higher in the GIS population. The influenza vaccination coverage in the GIS population is particularly higher in the last three seasons. This increased coverage is most likely associated with the increased participation of 60+-year-old volunteers, in which the influenza vaccination coverage is higher. The influenza vaccination coverage in the 65+ age group is slightly higher in the GIS population. The spatial population distributions of the two populations are similar.

**Table 2:** Characteristics of the GIS and Flemish population.

Characteristic	2003–04	2004–05	2005–06	2006–07	2007–08	2008–09	2009–10	2010–11	Flanders
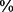 males	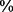 58.15	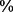 54.75	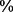 54.05	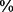 54.59	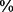 52.63	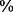 54.65	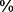 53.80	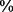 55.55	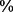 49.30
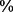 females	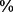 41.85	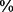 45.25	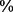 45.95	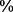 45.41	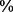 47.37	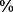 45.35	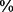 46.20	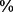 44.45	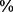 50.70
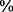 asthma	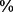 4.59	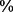 3.74	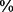 4.59	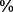 4.44	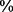 4.86	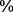 5.49	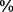 5.58	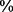 5.52	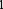 2.1–4.3 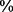
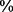 diabetes	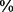 1.63	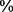 2.06	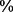 2.50	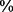 2.65	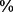 3.54	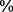 4.46	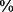 4.24	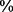 4.61	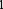 1.7–3.9 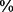
vaccination coverage	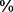 24.66	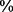 28.93	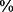 35.94	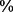 35.57	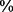 35.83	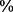 44.12	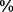 47.20	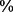 44.54	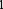 20.5–32.2 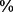
vaccinationcoverage 65+	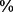 75.36	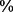 74.58	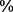 78.31	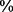 74.17	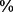 75.96	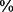 76.50	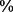 79.17	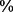 73.96	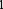 53.5–72.4 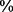
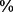 Antwerp	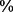 31.77	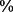 32.77	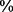 29.86	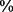 31.03	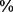 30.00	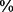 30.54	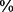 30.76	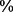 31.66	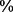 27.82
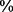 Limburg	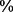 11.00	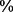 10.22	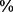 12.85	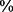 12.66	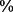 13.18	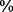 12.44	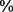 12.55	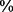 12.04	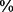 13.40
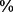 Vlaams-Brabant	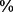 18.18	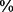 19.29	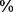 19.76	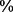 19.75	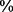 18.37	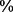 19.33	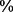 19.45	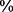 19.42	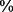 17.19
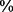 Oost-Vlaanderen	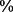 23.29	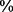 22.60	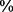 20.84	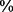 20.39	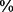 21.41	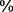 21.04	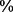 20.89	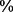 21.18	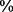 22.87
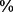 West-Vlaanderen	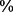 15.76	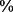 15.12	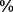 16.69	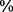 16.18	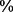 17.04	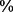 16.66	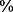 16.35	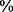 15.69	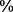 18.71

Some important demographic and medical statistics from the GIS population in each year are compared with the corresponding numbers in the Flemish population. The ‘Flanders’ column shows the average value of the corresponding characteristic during the eight years under study.

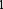
The range of the characteristic obtained from the 2001, 2004 and 2008 Health Interview Survey in Belgium.

### Validity of the GIS

**Figure 2:**
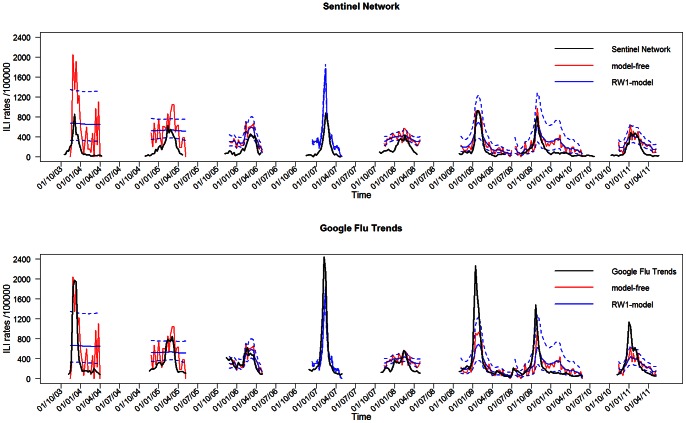
Estimated ILI incidence trend from the GIS. The estimated ILI incidence trends based on the GIS data (using the *restricted sample*) are shown together with the trends from the Sentinel Network (above) and Google Flu Trends (below). Incidence of ILI is shown per 100000 participants. The dashed lines of the *RW1-model* present the 95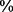 confidence interval of the estimated ILI incidence.

Figure 2 presents the estimated ILI trends using the *restricted sample*, together with the trends from the Sentinel Network and the Google Flu Trends. For the first two seasons, the *RW1-model* is not able to detect a trend amid the noise of the GIS data. The *RW1-model* approach estimates trends that are smoother than the *model-free* trends. Especially in the last three seasons, all surveillance systems show a comparable course over time. In the early years, the estimated ILI trends based on the GIS data show higher incidences than those of the Sentinel Network. This effect diminishes over time. In the later years, the heights of the incidence curves are similar. An opposite effect is observed for the Google Flu Trends incidence curves. For most seasons, a higher background level of ILI incidence for the GIS is apparent outside the peak periods. Although trends estimated by the *complete sample* yield higher incidence peaks, they are comparable in time with those from the *restricted sample* (not shown). This difference in height is most pronounced in the early years. This result can be expected, since in the early years a higher percentage of volunteers that participate only once is observed (Table 1).

Correlations between the estimated ILI incidence trends of the GIS and trends from the other two monitoring systems are satisfactory (Table 3). In the 2006–2007 season and the last three seasons, the correlations are highest. Ignoring the first two seasons, the *RW1-model* approach yields similar or higher correlations. In the later seasons, the *complete sample* performs at least as well as the *restricted sample* in terms of correlations with the other two monitoring systems. This result indicates that in the last seasons, the data of all participants can safely be used to estimate ILI trends from the GIS.

**Table 3:** Raw correlations between estimated ILI incidence from the GIS and two other monitoring systems.

Monitoringsystem	Model	2003–04	2004–05	2005–06	2006–07	2007–08	2008–09	2009–10	2010–11
	*Complete sample*								
Sentinel	*free*	0.77(0.63)	0.72(0.77)	0.60(0.62)	0.87(0.91)	0.65(0.64)	0.95(0.91)	0.86(0.60)	0.85(0.83)
	*RW1*	0.85(0.85)	0.71(0.74)	0.73(0.79)	0.88(0.91)	0.83(0.81)	0.94(0.89)	0.83(0.68)	0.95(0.90)
Google	*free*	0.92(0.81)	0.78(0.63)	0.75(0.79)	0.95(0.89)	0.72(0.65)	0.89(0.93)	0.73(0.88)	0.81(0.81)
	*RW1*	0.76(0.67)	0.75(0.74)	0.83(0.85)	0.95(0.89)	0.87(0.76)	0.86(0.91)	0.62(0.76)	0.85(0.88)
	*Restricted sample*								
Sentinel	*free*	0.69(0.66)	0.71(0.56)	0.79(0.79)	0.88(0.89)	0.59(0.59)	0.96(0.89)	0.78(0.52)	0.81(0.78)
	*RW1*	0.81(0.81)	0.90(0.78)	0.76(0.83)	0.88(0.89)	0.83(0.80)	0.94(0.87)	0.77(0.57)	0.94(0.87)
Google	*free*	0.82(0.63)	0.72(0.71)	0.74(0.77)	0.94(0.89)	0.67(0.60)	0.86(0.94)	0.62(0.81)	0.76(0.80)
	*RW1*	0.67(0.61)	0.87(0.81)	0.82(0.84)	0.94(0.89)	0.86(0.75)	0.84(0.91)	0.62(0.76)	0.82(0.87)

Raw correlations between the estimated ILI incidence trends based on the GIS data and the trends from the Belgian Sentinel Network and Google Flu Trends in Flanders. Cross-correlations with a lag time of one week (GIS *vs.* Sentinel Network 1 week later; GIS 1 week later *vs.* Google Flu Trends) are provided between the brackets. Cross-correlation are used to investigate whether there is a better correlation between two monitoring systems when one monitoring system is given a time lag (only the results of one week are provided because they yielded the highest cross-correlations). Results are shown for the analysis with the *model-free* and *RW1-model* approaches for both the *complete* and *restricted samples*.

Table 4 presents the relative risk results of the multivariate risk factor analysis using the *restricted sample* after model building. For the analysis based on the 2003 to 2011 influenza seasons, excluding the 2009–2010 H1N1 influenza season, belonging to a younger age group, being female, living with children, having chronic diseases, and daily smoking are found to be factors associated with an increased risk of having an ILI episode during an influenza season. No difference in gender is observed for the 0- to 12-year age group (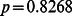). A significant difference in risk is observed between men and women for the 13- to 64-year age group, for those who live with children (95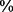 CI of RR: [0.72–0.96]), and for those who do not live with children in their household (95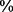 CI of RR: [0.68–0.85]). Men having children in their household have an increased risk over men not having children in their household (95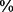 CI of RR: [1.22–1.62]). A similar result is found for women (95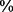 CI of RR: [1.15–1.46]). The results for the H1N1 pandemic influenza season are qualitatively similar (Table 4). Some factors do not show a significant effect on the risk of ILI, which could be explained by the smaller sample size. Similar results are obtained when using the *complete sample* (not shown).

**Table 4:** Results of the multivariate regression model for risk factor analysis.

2003–04 to 2010–11 (without 2009–10)			2009–10		
	RR [95 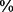 CI]	p-value		RR [95 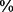 CI]	p-value
Demography			Demography		
0–12 y male	6.01 [3.24–11.16]	<0.0001	0–12 y male	11.52 [2.22–59.85]	0.0037
0–12 y female	5.73 [3.08–10.65]	<0.0001	0–12 y female	10.81 [2.22–52.52]	0.0032
13–64 y male (w children)	2.90 [1.68–5.01]	0.0001	13–64 y male (w children)	4.53 [1.09–18.80]	0.0375
13–64 y female (w children)	3.48 [2.02–6.01]	<0.0001	13–64 y female (w children)	6.79 [1.66–27.82]	0.0078
13–64 y male (w/o children)	2.04 [1.18–3.51]	0.0101	13–64 y male (w/o children)	3.04 [0.74–12.40]	0.1216
13–64 y female (w/o children)	2.69 [1.56–4.63]	0.0004	13–64 y female (w/o children)	5.03 [1.24–20.44]	0.0238
65+ y male	0.87 [0.47–1.60]	0.6490	65+ y male	1.00 [0.21–4.81]	0.9982
65+ y female	–		65+ y female	–	
Chronic diseases			Chronic diseases		
None	0.74 [0.64–0.86]	<0.0001	None	0.88 [0.60–1.29]	0.5036
Asthma/Diabetes/Both	–		Asthma/Diabetes/Both	–	
Smoking status			Smoking status		
Daily	1.30 [1.15–1.46]	<0.0001	Daily	1.64 [1.20–2.24]	0.0019
Sometimes	1.13 [0.95–1.35]	0.1770	Sometimes	0.83 [0.44–1.56]	0.5644
Don’t smoke	–		Don’t smoke	–	

Estimated risk ratios (RR) [95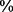 CI] of the risk factor analysis based on the *restricted sample*. We control for influenza vaccination status in the model. Results from the 2003–04 to 2010–11 influenza seasons (excluding the H1N1 pandemic influenza season of 2009–10) are shown on the left. The results from the 2009–10 influenza season are shown on the right. y: years; w: with; w/o: without.

## Discussion

The Great Influenza Survey started in 2003 in an attempt to monitor influenza-like illness in the general population via the internet. To determine the validity of the GIS, it is important to investigate the representativeness of the recruited population [26]. The age distributions of the GIS and Flemish population are dissimilar. Children and the elderly are clearly underrepresented in the GIS, which is likely associated with internet usage in these age groups. Over time, a marked increase in participation is observed for the 60- to 69-year age group, which is likely attributed to the growing internet usage in this age group [27]. Similar age distributions were also found in the GIS in the Netherlands [4], [5] and the Flusurvey in the UK [7]. Because of the small number of participants in some age categories (e.g., children and elderly), the GIS is not helpful in estimating the ILI incidence for specific age groups. This is, however, an important surveillance objective. Since the 2012–2013 influenza season, parents have been increasingly requested to also participate in the GIS on behalf of their children. The prevalence of asthma, diabetes, and influenza vaccination rates are in line with statistics in the general population. Thus, a high level of equivalence in health exists between the GIS and the Flemish population.

Despite the unrepresentativeness in terms of age, satisfactory correlations are found between estimated ILI incidence trends based on the GIS and trends obtained from the Sentinel Network and the Google Flu Trends. This indicates that valid incidence trends are obtained via the GIS. The GIS measures higher background levels of ILI incidence outside the peak periods. This was also observed in the Netherlands and the UK [5], [7]. These higher background levels are likely observed because GPs are probably more critical in making an ILI diagnosis outside the influenza peak periods. In the beginning years of the survey, the ILI incidence trends of the GIS are two to three times higher than those measured by the Sentinel Network. This difference diminishes over time and disappears in the last years. The fact that fewer individuals participated in the GIS as a one-off response to their symptoms in later years, is a possible explanation for this result. By contrast, in the Netherlands, there is a 10∶1 ratio between the height of the GIS and the Sentinel Network trends and this ratio remained stable over time [5]. The difference between Flanders and the Netherlands likely results from two reasons. First, in the Sentinel Network, a 6∶1 ratio is observed between the Belgian and Dutch data. However in the GIS, similar heights are observed for Belgium and the Netherlands. Second, health-seeking rates for participants matching the ILI case definition are different. In the Netherlands between 20 and 25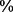 of the participants visited a GP [5], [6], while in the Flemish GIS, this percentage is about 60–70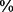.

Ignoring the first two seasons, the *RW1-model* approach to estimate ILI incidence trends works well. This approach has the extra advantage of estimating the variability associated with the incidence trends.

Belonging to a young age group, living with children, being female, having asthma and/or diabetes and daily smoking were all found to be risk factors associated with an increased risk of having an ILI episode. These results are consistent with the ILI literature [28]–[37], which further validates the use of the GIS. Women have an increased risk compared to men, possibly because they often spend more time near children. Because the GIS is an observational study and the data exhibit high variation, VE estimates with wide confidence intervals are obtained and are therefore not reported. Extracting valid VE estimates from the GIS in Flanders, in light of the work of Eames and colleagues [28] in the UK, is a topic of further research.

Regarding trend estimation, almost no difference is observed between the *complete* and *restricted sample* in the later years. The risk factor analysis also yields similar results between both samples. These results show that one can use the information from all participants in the later years. This is in contrast to the UK Flusurvey where it was found that people entering the survey because of their symptoms bias the results [7]. However, these results are based on the first year the Flusurvey was held in the UK. In the first years of the Flemish GIS is also found that these non-regular participants distort the results, but this distortion diminishes over time. In the later years, this distortion vanishes and the *complete* sample can safely be used.

The Belgian Sentinel Network is the best established ILI surveillance network in Belgium and yields important information with regard to virology and incidences in different age groups. We regard the GIS as a useful and promising surveillance system to monitor ILI that adds information to the existing surveillance systems. Indeed, the fact that individual data on demographics, lifestyle and medical status are available is helpful in detecting factors that influence the ILI burden (see, for example, Smolderen *et al.* [38]). The symptom questionnaire contains more symptoms than are needed to define ILI, which makes the survey also useful for studying other syndromes. The GIS assesses the ILI incidence level more rapidly as does Google Flu Trends when compared to the traditional Sentinel Network, because the information of this last monitoring system is only available with a one-week delay. The GIS does not require individuals to seek health care and therefore likely captures a wider range of cases. Especially in case of a pandemic, when the health care system is under stress and medical care seeking rates are different, internet-based ILI monitoring has proven to be useful because it monitors ILI directly from the community [7]. Drawbacks of the GIS are the fact that the GIS population is self-selecting, no indication of misreporting is available, and no virological data are available.

In conclusion, although the GIS is unrepresentative in terms of the age distribution, the ILI incidence trends estimated from the GIS data correlate well with the Sentinel Network and the Google Flu Trends data. The GIS does not offer a substitute for the traditional surveillance system by the Sentinel Network of GPs, but it can be an important complementary monitoring system that is timelier, offers data at the individual level and measures the ILI incidence directly in the community. If there is a difference in trend between the monitoring systems it means that we are missing something in one of the systems and we could learn from this.

## Supporting Information

Text S1 (PDF)
