# Supplementary material for: Eight Years of the Great Influenza Survey to Monitor Influenza-Like Illness in Flanders
Source: PLoS One. 2013 May 17;8(5):e64156. doi: 10.1371/journal.pone.0064156 (PMC3656949; doi:10.1371/journal.pone.0064156)
Supplement: Text S1 — (PDF) [file pone.0064156.s001.pdf]

Supporting information for

Eight years of the Great Influenza Survey to monitor influenza-like illness in Flanders

Yannick Vandendijck[1,*], Christel Faes[1], Niel Hens[1,2]

1 Interuniversity Institute for Biostatistics and statistical Bioinformatics, Hasselt University, Diepenbeek, Belgium
2 Centre for Health Economic Research and Modeling Infectious Diseases, Vaccine and Infectious Disease Institute, University of Antwerp, Wilrijk, Belgium
∗ E-mail: yannick.vandendijck@uhasselt.be

In this supporting information, more details on the random walk model of first order (*RW1-model*) are given. The model is explained, and choices of the prior distribution for all parameters can be found.

Random Walk Model of First Order

Assume that in week i the number of new ILI cases y_i is negatively binomial distributed, $y_i \sim \text{NegBin}(\alpha_i, \tau)$. The τ is often referred to as the dispersion parameter. The mean and variance of y_i are $\mu_i = \tau(1-\alpha_i)/\alpha_i$ and $\sigma_i^2 = \mu_i(1 + \mu_i/\tau)$, respectively. Assume that the mean μ_i is modeled as $\mu_i = E_i \exp(\eta_i)$, where E_i represents the known offset in week i and η_i is a linear predictor. The offset E_i is chosen equally to the number of active participants in week i. The linear predictor η_i is modeled as $\eta_i = \beta_0 + \delta_i$, where β_0 represents the intercept and $\boldsymbol{\delta} = (\delta_1, ..., \delta_T)'$ are time-specific parameters that follow a random walk model of first order. The latter model assumes that the differences between two adjacent parameters follow a multivariate normal distribution with a mean of zero. This results in the following distribution for $\boldsymbol{\delta}$ [1]:

$$\begin{aligned}\pi(\boldsymbol{\delta}|\sigma_\delta^2) &\propto \exp\left(-\frac{1}{2\sigma_\delta^2}\sum_{t=2}^{T}(\delta_t - \delta_{t-1})^2\right)\\ &= \exp\left(-\frac{1}{2\sigma_\delta^2}\boldsymbol{\delta}'\mathbf{R}_\delta\boldsymbol{\delta}\right),\end{aligned}$$

where σ_δ^2 is an unknown variance parameter and $\mathbf{R}_\delta$ is a structured matrix of the form given by

$$\mathbf{R}_\delta = \begin{pmatrix} 1 & -1 & & & & & \\ -1 & 2 & -1 & & & & \\ & -1 & 2 & -1 & & & \\ & & \vdots & \vdots & \vdots & & \\ & & & -1 & 2 & -1 & \\ & & & & -1 & 2 & -1 \\ & & & & & -1 & 1 \end{pmatrix}.$$

A Bayesian approach is taken, and the following uninformative priors are chosen for the unknown parameters:

$$\begin{aligned}\log(\sigma_\delta^2) &\sim \text{LogGamma}(1, 0.001),\\ \pi(\beta_0|\nu_1) &\sim \mathcal{N}(0, \nu_1^{-1}) \quad \text{with } \nu_1 = 0.001,\\ \pi(\tau|\nu_2) &\sim \mathcal{N}(0, \nu_2^{-1}) \quad \text{with } \nu_2 = 0.001.\end{aligned}$$

This model is fitted using approximate Bayesian inference by integrated nested Laplace approximations (INLA) [2]. This method yields very good approximate Bayesian inference in structured additive regression models with latent Gaussian fields. A major advantage of INLA is that it returns accurate parameter estimates in a short computational time. Models were fit in `R` version 2.14 using the `INLA` package [3].

References

1. Schrödle B, Held L (2011) Spatio-temporal disease mapping using INLA. Environmetrics 22: 725-734.

2. Rue H, Martino S, Chopin N (2009) Approximate Bayesian inference for latent Gaussian models by using integrated nested Laplace approximations (with discussion). J Roy Stat Soc B 71: 319-392.

3. Martino S, Rue H (2009) Implementing approximate Bayesian inference using integrated nested Laplace approximation: a manual for the INLA program. Available from: `http://wwwr-inlaorg/download` .
